# Patient-specific 3D *in vitro* modeling and fluid dynamic analysis of primary pulmonary vein stenosis

**DOI:** 10.3389/fcvm.2024.1432784

**Published:** 2024-07-04

**Authors:** Christian Devlin, Martin L. Tomov, Huang Chen, Sindhu Nama, Siraj Ali, Sunder Neelakantan, Reza Avazmohammadi, Lakshmi Prasad Dasi, Holly D. Bauser-Heaton, Vahid Serpooshan

**Affiliations:** ^1^ Wallace H. Coulter Department of Biomedical Engineering, Emory University School of Medicine and Georgia Institute of Technology, Atlanta, GA, United States; ^2^ Department of Biomedical Engineering, Texas A&M University, College Station, TX, United States; ^3^ J. Mike Walker ‘66 Department of Mechanical Engineering, Texas A&M University, College Station, TX, United States; ^4^ School of Engineering Medicine, Texas A&M University, Houston, TX, United States; ^5^ Department of Pediatrics, Emory University School of Medicine, Atlanta, GA, United States; ^6^ Children’s Healthcare of Atlanta, Atlanta, GA, United States; ^7^ Sibley Heart Center, Children’s Healthcare of Atlanta, Atlanta, GA, United States

**Keywords:** 3D printing, pulmonary vein stenosis (PVS), flow hemodynamics, particle image velocimetry, computational fluid dynamics, stenting, cardiac intervention

## Abstract

**Introduction:**

Primary pulmonary vein stenosis (PVS) is a rare congenital heart disease that proves to be a clinical challenge due to the rapidly progressive disease course and high rates of treatment complications. PVS intervention is frequently faced with in-stent restenosis and persistent disease progression despite initial venous recanalization with balloon angioplasty or stenting. Alterations in wall shear stress (WSS) have been previously associated with neointimal hyperplasia and venous stenosis underlying PVS progression. Thus, the development of patient-specific three-dimensional (3D) *in vitro* models is needed to further investigate the biomechanical outcomes of endovascular and surgical interventions.

**Methods:**

In this study, deidentified computed tomography images from three patients were segmented to generate perfusable phantom models of pulmonary veins before and after catheterization. These 3D reconstructions were 3D printed using a clear resin ink and used in a benchtop experimental setup. Computational fluid dynamic (CFD) analysis was performed on models *in silico* utilizing Doppler echocardiography data to represent the *in vivo* flow conditions at the inlets. Particle image velocimetry was conducted using the benchtop perfusion setup to analyze WSS and velocity profiles and the results were compared with those predicted by the CFD model.

**Results:**

Our findings indicated areas of undesirable alterations in WSS before and after catheterization, in comparison with the published baseline levels in the healthy *in vivo* tissues that may lead to regional disease progression.

**Discussion:**

The established patient-specific 3D *in vitro* models and the developed *in vitro*–*in silico* platform demonstrate great promise to refine interventional approaches and mitigate complications in treating patients with primary PVS.

## 1 Introduction

Primary pulmonary vein stenosis (PVS) is a pediatric disease of the congenital pulmonary vasculature with a prognosis that remains poor despite recent advances in endovascular and surgical techniques (1–3). The 5-year survival rate of PVS remains below 50% due to frequent in-stent restenosis and progression of the disease despite intervention (4–6). Patients with congenital PVS exhibit significant disease heterogeneity owing to the variance in the number of occluded veins, degree of stenosis, and other comorbid conditions that affect cardiac anatomy and hemodynamics (7). Although the onset of primary PVS is thought to have an embryologic origin, alterations in wall shear stress (WSS) complicated by a tortuous anatomical course are likely an important driver of disease progression (8). Prior studies have demonstrated that decreased levels of WSS lead to pathological vascular changes, notably the dedifferentiation of endothelial cells to undergo a mesenchymal transition (9). Similar alterations in WSS have been previously associated with coronary plaque formation and stent restenosis (10, 11). In PVS, neointimal hyperplasia and fibrosis are primary drivers of disease progression and have been associated with disturbed venous WSS (12). Considering these challenges, there is a significant need to investigate the hemodynamic and biomechanical alterations underlying PVS in a patient-specific manner to guide future clinical management. In this study, we developed patient-specific, 3D printed, perfusable *in vitro* phantoms and *in silico* models of PVS in the pre- and post-catheterization states. We employed complementary techniques in computational fluid dynamics (CFD) modeling and particle image velocimetry (PIV) to analyze the hemodynamic and biomechanical outcomes of endovascular intervention.

The etiology of primary PVS is thought to be a failed embryologic connection of the left atrial outpouching with the confluence of fetal pulmonary veins, resulting in significant fibrosis or atresia of one or more pulmonary veins (7, 8, 13). Patients often initially present in the first year of life with non-specific symptoms of tachypnea and recurrent pulmonary infections (13). Disease progression often leads to signs and symptoms of pulmonary hypertension, including hemoptysis, right ventricular overload, and cardiac murmurs (2, 14). Diagnosis and management of primary PVS is frequently challenged by coexisting congenital cardiac malformations, seen in up to 50% of patients with primary PVS, which complicate disease management and interventional approaches (8). Catheter-based interventions of PVS, including balloon angioplasty (15) and endovascular stenting (16) in PVS, have historically been burdened with significant rates of restenosis and pulmonary regurgitation (5, 17). The recent increase in the use of drug-eluting stents has shown initial promise for improving the rates of in-stent restenosis (18); however, stent placement is not always feasible due to severe stenosis or venous anatomy (7). Modern surgical approaches involve resection, mobilization, reorientation, and anastomosis of the pulmonary veins (19). Many of these novel surgical techniques, however, also require further investigation to understand the altered anatomy and blood flow (20).

To date, significant advances in additive manufacturing, and in particular, 3D printing technologies, such as extrusion, stereolithography, and digital light processing (DLP)-based printing, have substantially improved the ability to develop highly accurate *in vitro* models of various tissues and organs (21–24). 3D printed models provide unparalleled capacity to recapitulate the highly complex and dynamic microenvironment of various tissues and, therefore, have found increasing applications in modeling a variety of congenital heart disease (25–27). The creation of patient-specific anatomical 3D models has been shown to be a greatly effective tool in cardiac interventional planning and medical education (28). Notably, techniques in additive manufacturing and fluid dynamic analysis have been previously used to study pulmonary artery atresia (PAA) and tetralogy of Fallot (TOF) with major aortopulmonary collateral arteries (MAPCAs) by the creation of simplified perfusable phantom models and subsequent CFD and PIV analyses (29). In PVS, a similar approach can be employed using 3D-segmented models from patient imaging of the congenital pulmonary veins.

Computational and experimental analyses of flow hemodynamic parameters within the 3D congenital vasculature have been proven to be robust tools in examining the impact of flow (alterations) on various congenital heart diseases (30–33). CFD analyses employ differential equations and numerical methods to predict fluid behavior using *in silico* models, based on fundamental equations that describe conservation of momentum and mass. Computational modeling has been shown to be instrumental in modeling various cardiovascular pathologies, including various aortic anomalies, Norwood anatomy, single-ventricle defects, and PAA (34–37). CFD modeling has also been shown to be useful in interventional and surgical planning to study hemodynamic effects of stenting and compare surgical approaches (38). Similarly and in parallel to the *in silico* models, *in vitro* PIV methods have been proven to be a valuable experimental technique to study flow characteristics in cardiovascular models (29, 39, 40). Through the use of laser or ultrasound-based approaches, PIV techniques have enabled precise tracking of flow within the 3D geometries and quantifying fluid flow parameters such as planar and 3D WSS and velocity vectors, which are critical factors in the pathogenesis of congenital pulmonary vascular disease (29, 34, 41).

## 2 Experimental methods

### 2.1 CT imaging, segmentation, and creation of 3D perfusable models

Deidentified computed tomography (CT) angiography (CTA) data were taken from patients with congenital PVS in the setting of idiopathic, single ventricle, and totally anomalous pulmonary venous return pathologies. CTA images were loaded as DICOM files using 3D Slicer (Brigham and Women's Hospital, Boston, MA, USA) software for segmentation (42, 43). Five sets of pulmonary veins and attached left atrium pre- and post-catheterization data were segmented for these three patients. Manual segmentation was performed on each geometry. Primary anatomical branches were segmented for each pulmonary vein. Stereolithography (STL) files were exported to Ansys SpaceClaim (Images used courtesy of Ansys® Inc., SpaceClaim 2022 R2, Lebanon, NH, USA) for 3D model post-processing and perfusion engineering. An outer shell with 1mm wall thickness was created for each geometry to perform benchtop and PIV flow testing. Perfusion inlets and outlets were created to connect to a peristaltic pump for benchtop flow testing. Inlet barbs [2.25 mm internal diameter (ID) × 20 mm length] were designed for each anatomical inlet to decrease turbulence created by the inlet tubing interface in benchtop and CFD testing. A single outlet connection was created and attached to the left atrium to simulate the mitral outlet (12 mm ID × 5 mm length). These computer-aided design (CAD) models with varying degrees of stenosis were 3D printed on the Form 3+ stereolithography 3D printer (Formlabs, Somerville, MA, USA) using a 25 µm layer thickness. Each model was printed using external supports and underwent recommended post-processing. After printing, each model was washed for 60 min in 100% isopropyl alcohol to remove non-crosslinked resin from the models and subsequently underwent a 25-min curing stage at 60 °C UV for final processing of the resin.

### 2.2 Benchtop perfusion and PIV setup

#### 2.2.1 Creation of polydimethylsiloxane constructs and benchtop perfusion setup

To utilize laser-based PIV technique for the analysis, 3D constructs of optically transparent polydimethylsiloxane (PDMS) (Sylgard™ 184 Silicone Elastomer Kit, Dow, CA, USA) were created for pre- and post-catheterization models for the left lower pulmonary vein (LLPV) from patient #1 with idiopathic PVS. Perfusable inner-shelled resin models were created with a 0.6 mm wall thickness and were 3D printed and used as model molds for PDMS casting. Molding boxes were 3D printed using a filament extrusion printer, and holes were created to allow for inlet and outlet tubing connections. Perfusion attachments were connected to the model inside the molding boxes, and the boxes were sealed using 100% silicone rubber. A 10:1 ratio of PDMS to crosslinking agent was used for curing. Vacuum aspiration was performed on crosslinking PDMS for 30 min to remove air bubbles before pouring. PDMS was poured into the molding boxes and was initially cured at 80 °C for 2 h. The models were then left at room temperature (20 °C) for 48 h to complete curing. Once cured, the models were perfused with 100% acetone for 4 days to fully clear the inner 3D resin models, which did not undergo a UV curing in post-processing. The resulting PDMS block was extracted from the molding box, and the imaging surfaces were leveled. Diameter-matched inlet and outlet silicone tubing was used to connect the PDMS models to a 12 V variable speed peristaltic pump (Kamoer©, Kamoer Fluid Tech Co., Shanghai, China) set to an average flow rate of 79 ml/min, which was calculated using the LLPV post-catheterization average velocity from pulse–Doppler echocardiogram data and the cross-sectional lumen area.

#### 2.2.2 PIV experimental setup

A 2D laser PIV assay was constructed to image and quantify flow through the LLPV PVS model using a benchtop perfusion setup. The PDMS block was submerged in an index-matching aqueous urea solution with a refractive index of 1.41. A water–glycerol–urea mixture at a density of 1,130 kg/m^3^ and a viscosity of 0.0039 kg/(m s) in the range of blood viscosity was used for the perfusion setup. We added 2 µm tracer polystyrene particles, coated with Rhodamine 6G (LaVision 1001851), to the solution and illuminated them during the experiments using a double-pulse from a Nd:YLF laser (DM20-527-DH, Photonics Lasers, Ronkonkoma, NY, USA). A Phantom VEO-E 340l (AMETEK, Berwyn, PA, USA) camera with a high-speed sensor was used with a Nikon (Melville, NY, USA) NIKKOR Z MC 105 mm F 2.8 VR S Macro lens for imaging flow through a mirror setup. The laser sheet was oriented to the image along the longitudinal plane including the stenosis/stented regions as well as a distal region. The images were recorded for multiple peristaltic pump cycles and velocity vectors were calculated. Vector data were post-processed using ParaView (Kitware, Clifton Park, NY, USA).

### 2.3 CFD analysis of flow hemodynamics

CFD analysis was performed on the pre- and post-catheterization idiopathic LLPV models. In Ansys SpaceClaim, the interior fluid volumes of the perfusable models were used for CFD testing. One outlet and two inlet boundaries were used for CFD testing. The meshes were generated using tetrahedral mesh elements. A transient analysis was performed on these models using Ansys SpaceClaim, Meshing, and Fluent. Two CFD tests were performed on each geometry, one to compare with the results of PIV and another to best mimic *in vivo* conditions. Planes of interest were chosen for the analysis. These included a plane in the distal pulmonary vein, a plane through the stenotic and subsequently stented region, as well as a longitudinal plane in the PIV experiments.

#### 2.3.1 PIV simulations

For PIV comparison, a water–glycerol–urea mixture was simulated in Fluent with a density of 1,130 kg/m^3^ and a viscosity of 0.0039 kg/(m s), which were included under a Newtonian fluid assumption. The flow rate vs. time input function was determined from flow measurements performed on the peristaltic pump used in the benchtop flow setup. The same input function was used for both inlets of the LLPV model.

#### 2.3.2 Simulating the *in vivo* flow conditions

A set of CFD simulations was conducted using parameters to best match *in vivo* conditions. A density of 1,060 kg/m^3^ was chosen for this analysis, and given the non-Newtonian viscosity characteristics of blood, a Casson fluid model was chosen to model the variable blood viscosity with respect to shear rate as follows:

$$\mu =\frac{\mu_{\infty }^{2}}{\dot{\gamma }}+\frac{2\mu_{\infty }N_{\infty }}{\sqrt{\dot{\gamma }}}+N_{\infty }^{2}$$

$$N_{\infty }=\sqrt{\mu_{p}{\left(1-\mathrm{Hct}\right)}^{-0.25}}$$

$$\mu_{\infty }=\sqrt{0.625\mathrm{Hct}}$$

where μ is the viscosity, $\dot{\gamma }$ is the shear rate, $\mu_{\infty }^{2}$ is the yield stress, $N_{\infty }$ is the consistency index, and Hct is the blood hematocrit. For the blood, $\mu_{p}=0.00145$ (Pa s) and Hct=0.4.

Pulse–Doppler echocardiogram data were obtained from pre- and post-catheterization studies. The post-catheterization Doppler waveforms were curve fitted and used as the velocity vs. time profiles for the CFD analysis. Gravity was simulated on the geometry assuming the patient was in a vertical, standing position. The *in vivo* simulations were conducted over one cardiac cycle using a fixed time step, and convergence was verified. A time point at the systolic peak of the pulse Doppler waveform was chosen to observe WSS and velocity levels in the PVS models.

#### 2.3.3 CFD solution parameters

In both sets of CFD simulations, a parabolic inlet function was described as a user-defined function to simulate fluid flow. A pressure-based solver was chosen for the analysis. A no-slip wall condition was applied, and a coupled Green-Gauss cell-based solver was used for simulation. A turbulent two-equation k-epsilon flow model was used for the analysis. The results were generated using Ansys Fluent (Images used courtesy of Ansys® Inc. Fluent 2022 R2, Lebanon, NH, USA) and subsequently post-processed using ParaView.

## 3 Results

### 3.1 Creation of 3D printed, patient-specific, perfusable phantom models

**Figure 1:**
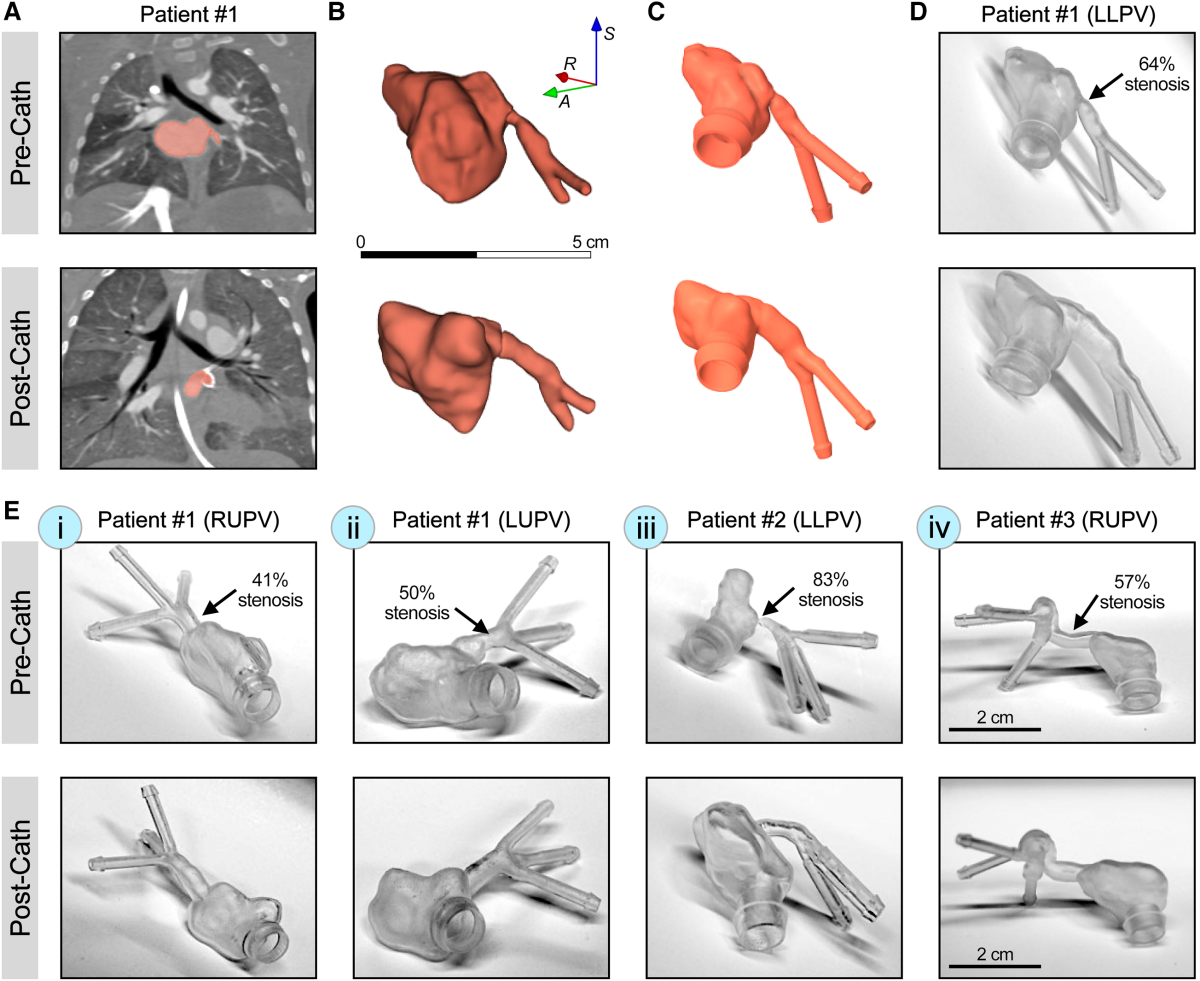
3D printing of patient-specific synthetic models of PVS. (**A**) CT scans with contrast from patient #1 with idiopathic PVS, demonstrating LLPV pre- (top) and post-catheterization (bottom). (**B**) Raw segmentations of LLPV and attached left atria. (**C**) The CAD model of LLPV with engineered inlet and outlet ports to reduce interface turbulence. (**D**) Form 3+ 3D printed resin models of patient #1 demonstrating stenosis in LLPV. (**E**) 3D printed resin models of stenosis in patient #1, in the RUPV (i) and the LUPV (ii), as well as in patient #2 (LLPV, iii) and patient #3 (RUPV, iv), with total anomalous pulmonary venous return. Scale bar in (**B**) shows 5 cm and that in (**E-iv**) shows 2 cm. Percentages of stenosis are shown for each condition in the pre-catheterization state.

In this study, five pulmonary vein models from the three patients were segmented and 3D printed (Figure 1). Pre-catheterization lumen diameters, degree of stenosis, and post-catheterization lumen diameters were measured using CTA data from each patient (Supplementary Figure S1, Supplementary Table S1). For patient #1, with idiopathic PVS, three pulmonary veins underwent catheterization between studies. Pre- and post-catheterization models were segmented and post-processed using 3D Slicer and Ansys SpaceClaim for the LLPV, right upper pulmonary vein (RUPV), and the left upper pulmonary vein (LUPV) (Figures 1D,E-i,ii). Three branching veins were included in the models for the RUPV and the LUPV. The LLPV was found to only have two identifiable branches on CTA. The LLPV, RUPV, and LUPV had 64%, 41%, and 50% stenosis, respectively, in the pre-catheterization imaging (Figures 1D,E-i,ii). For the second patient (patient #2) with PVS in the setting of single-ventricle shunt pathology, the LLPV was stented between CTA images and was chosen for the analysis with three branching inlet veins. This patient was found to have a high degree of stenosis of 83% (Figure 1E-iii). For patient #3, with PVS in the setting of total anomalous pulmonary venous return (TAPVR), the RUPV underwent surgical correction and was also included for the analysis with a 57% stenosis (Figure 1E-iv). Three inlet branches were identified and included in the models. The 3D resin models were 3D printed for all models using clear resin on the Formlabs Form 3+. The LLPV for patient #1 was chosen for the CFD and PIV analysis. Given such a high degree of stenosis in the RUPV in patient #2, the stenosis region was resolution-limited on CT imaging, so the model with the next highest degree of stenosis with adequate resolution was chosen.

### 3.2 PIV analysis of flow in patient #1—stenosis in LLPV

**Figure 2:**
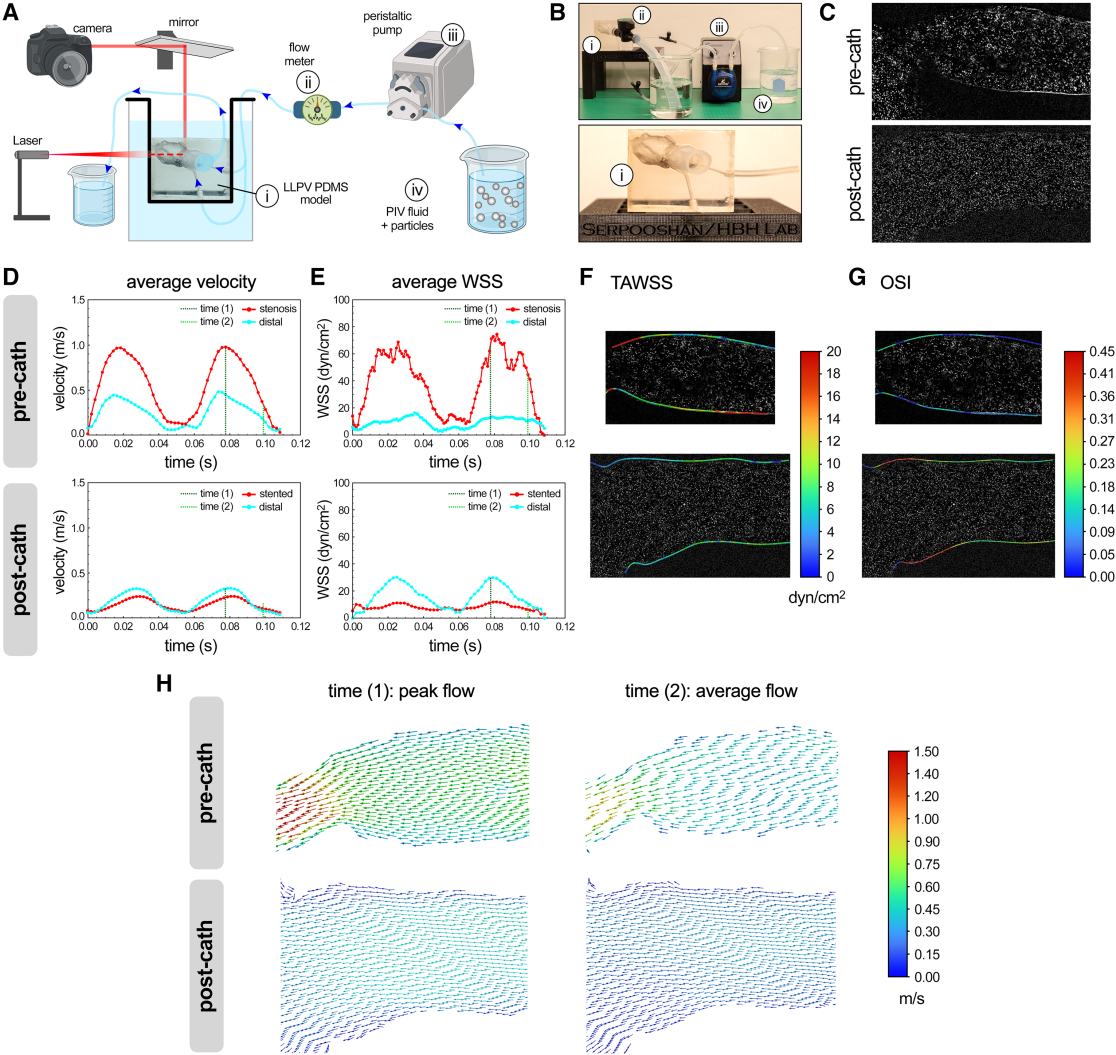
PIV analysis of flow hemodynamics in 3D printed models of PVS. Schematic (**A**) and actual (**B**) experimental setup used for the PIV analysis (partly created using Biorender.com). Key experimental components included a PDMS model of the LLPV (i), a flow meter (ii), peristaltic pump (iii), and a reservoir containing PIV fluid with particles (iv). Bottom panel in (**B**) shows the close-up view of the PDMS model of LLPV with two inlets and one outlet. (**C**) Imaging planes used for PIV analysis, pre-catheterization (top), and post-catheterization (bottom). Average velocity (**D**) and WSS (**E**) measured via PIV technique for two cycles of flow through the LLPV constructs pre- (top) and post-catheterization (bottom). 2D contours of TAWSS (**F**) and OSI (**G**). (**H**) Velocity vectors along the longitudinal plane at time (1) = peak systole (left) and time (2) = average flow (right), pre- (top) and post-catheterization (bottom).

A 2D laser PIV analysis was performed on the LLPV PVS model (patient #1, Figures 1A–D) using a benchtop perfusion setup (Figures 2A–C). Average velocity and WSS as functions of time during two flow cycles of the peristaltic pump were acquired through stenosis and distal planes (Figures 2D,E). PIV results in the pre-catheterization models demonstrated markedly greater (>2×) velocity and WSS values on the stenosis plane compared with that of the distal plane. Stenting of the LLPV resulted in a significant decrease in velocity and WSS, with values lower than those measured in the distal plane (Figures 2D,E, bottom). Furthermore, 3D time-averaged WSS (TAWSS) was calculated for a peristaltic pump cycle as follows:

$$\mathrm{TAWSS}=\frac{1}{T}\overset{T}{\underset{0}{\int }}|\mathrm{WSS}|\mathrm{dt}$$

Areas of elevated TAWSS were observed in the stenosis region with the smallest lumen diameter (Figure 2F). The oscillatory shear index (OSI) is a frequently used parameter in cardiovascular CFD analysis that describes local changes in the magnitude and size of WSS. High OSI has been associated with aberrant endothelial response (44) and is given as follows:

$$\mathrm{OSI}=\frac{1}{2}\left(1-\frac{\left|\int_{0}^{T}\mathrm{WSS}\;dt\right|}{\int_{0}^{T}|\mathrm{WSS}|dt}\right)$$

The highest levels of OSI were noted in the post-catheterization model near the pulmonary venoatrial junction (Figure 2G). In the next step, two time points during a pump cycle were included as points of interest for the simulation. The time (1) (=0.08 s) was chosen at the peak flow of the peristaltic pump of 158 ml/min and time (2) (=0.10 s) was at the average flow rate of 79 ml/min. Velocity vector data were generated along the longitudinal plane at times (1) and (2) (Figure 2H). The highest average velocities were visualized at time (1) through the stenosis region and reached 1.0 m/s.

### 3.3 Computational analysis of flow in patient #1—stenosis in LLPV

**Figure 3:**
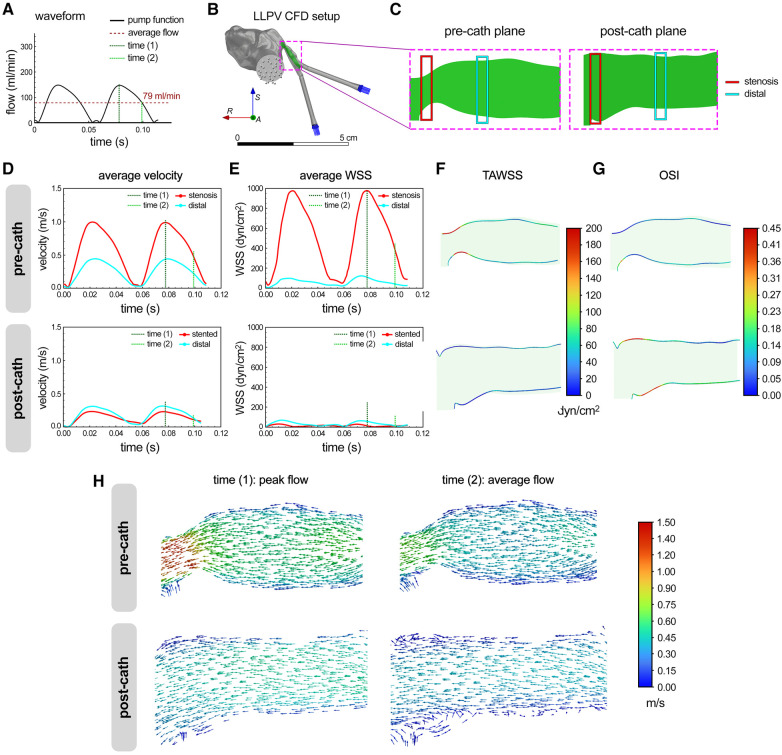
CFD modeling of flow hemodynamics in 3D printed models of PVS. (**A**) Flow waveform measured for the peristaltic pump used in the study for perfusion assays. This waveform was fed into the CFD model as an inlet function. (**B**) The setup used for the CFD analysis of flow through the LLPV PVS model, consisting of two inlets (blue arrows) and one outlet (gray arrows). The scale bar shows 5 cm. (**C**) Imaging planes used for the particle image velocimetry assay were replicated in CFD model before and after catheterization. Average velocity (**D**) and WSS (**E**) predicted via CFD during two peristaltic pump cycles, for flow through the LLPV PVS geometry pre- (top) and post-catheterization (bottom). 2D contours of TAWSS (**F**) and OSI (**G**). (**H**) Velocity vectors along the longitudinal plane, predicted at time (1) = peak systole (left) and time (2) = average flow (right), pre- (top) and post-catheterization (bottom).

The CFD modeling results were generated using the flow and rheological parameters consistent with those used in the PIV experiments. The same time points during the peristaltic pump cycle, time (1) (0.08 s) and time (2) (0.10 s), were used for the CFD analysis, representing the peak and average flows, respectively (Figure 3A). Further, the longitudinal planes were chosen to match those imaged with the laser sheet in the PIV experiments (Figures 3B,C). The average velocity and WSS were plotted as functions of time at the stenosis and distal planes in the pre- and post-catheterization models (Figures 3D,E). Consistent with the experimental (PIV) measurements (Figures 2D,E), the CFD model predicted significantly greater (>2×) levels of velocity and WSS in the stenosis plane compared with the distal plane. The stenting of the LLPV resulted in a drastic decrease in the velocity and WSS (Figures 3D,E, bottom). The highest velocities were predicted at time (1), through the stenosis region, reaching 0.98 m/s. 3D contours of TAWSS and OSI were also calculated for pre- and post-catheterization models (Figures 3F,G). Regions with the highest TAWSS were located in the stenotic and bifurcation regions of the pre-catheterization model, and regions with the highest OSI were in the distal region of the post-catheterization model, also at the venoatrial junction. Longitudinal planar vector fields were further constructed to demonstrate flow profiles through the stenosis and stented regions to match those obtained using the PIV (Figure 3H). Consistent with the PIV results (Figure 2H), the longitudinal vectors showed the highest velocity through the stenosis region.

**Figure 4:**
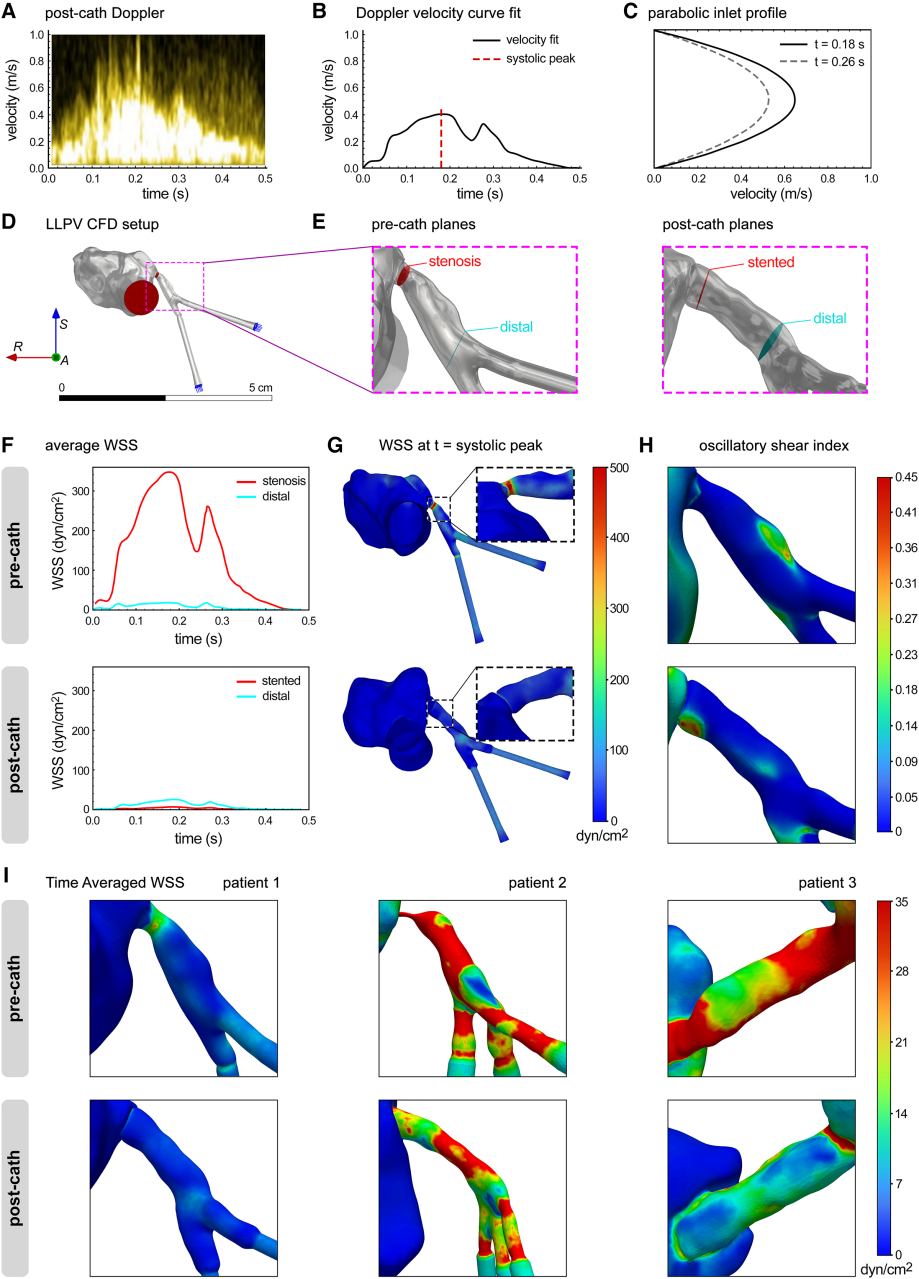
CFD modeling of flow hemodynamics in the clinical cases of PVS. (**A**) Post-catheterization Doppler echocardiography of LLPV during a single cardiac cycle. (**B**) Velocity vs. time curve fit was used to determine inlet flow rate function for CFD. (**C**) Parabolic user-defined inlet function in Ansys Fluent. (**D**) The LLPV setup used for CFD, with inlets (blue arrows) and outlet (red arrows). (**E**) Stenosis, stented, and distal planes in the pre-catheterization (left) and post-catheterization (right) models. (**F**) Average WSS vs. time in the pre-catheterization (top) and post-catheterization (bottom) models. (**G**) 3D contours of WSS at the *t* = systolic peak. (**H**) 3D contours of OSI. (**I**) 3D contours of TAWSS for patient #1 (anterior view), patient #2 (left view), and patient #3 (posterior view).

Next, CFD modeling was used to analyze the *in vivo* flow data in the reference LLPV PVS case (patient #1). The inlet flow rate vs. time profiles from patient echocardiogram data were verified in Ansys Fluent at each inlet plane (Figures 4A–C). The average WSS was calculated at distal and stenotic regions during one cardiac cycle. The average WSS at peak systole in the stenotic region was calculated at 346 dyn/cm^2^ (Figures 4D–F). In the post-catheterization model, WSS decreased to 6 dyn/cm^2^ in the stented region (Figure 4F). The 3D contours of WSS were generated at peak systole (Figure 4G). Regions of highest instantaneous WSS, as expected, were noted in the pre-catheterization model at the stenosis region with the smallest lumen size. In the post-catheterization models, highest levels of instantaneous WSS were also visualized at the bifurcation region. The highest levels of OSI were seen at this distal region in the pre-catheterization LLPV model and in the stented region of the post-catheterization model (Figure 4H). Contours of TAWSS were generated and scaled to an average WSS (35 dyn/cm^2^) above which venous neointimal hyperplasia and fibrosis have been shown to occur (Figure 4I) (12). In patient #1, elevated regions of TAWSS were present in the stenosis region as well as the distal and bifurcation regions before catheterization. In the post-catheterization models, TAWSS returned to healthy levels at the venoatrial junction; however, distal regions in the stent continued to experience elevated levels of TAWSS above a conservative threshold of 20 dyn/cm^2^ (Figure 4I). The CFD results from patient #2 indicated elevated levels of TAWSS in the severe stenosis region as well as in the distal branch vessels. Intervention decreased TAWSS in the venoatrial junction, but TAWSS remained high in the stented region. For patient #3, elevated levels of TAWSS were visualized throughout the pre-catheterization pulmonary vein. After surgical intervention, the levels of TAWSS were decreased in several regions but remained high in the region of large venous curvature. Furthermore, WSS levels remained elevated above healthy physiologic levels in the bifurcation region after stenting.

## 4 Discussion

Recent advancements in surgical and endovascular techniques have sought to alleviate the high mortality and morbidity rates of primary PVS (1). Despite the increasing clinical use of these novel therapies, patients with PVS still face high intervention complication rates. Given the significant disease heterogeneity in PVS and the variety of available interventional approaches, management of this life-threatening disease should be driven in a patient-specific fashion. 3D modeling, 3D printing and bioprinting, PIV, and CFD techniques address this challenge and shed light on patient-specific factors that may better predict interventional outcomes in PVS. In this study, we utilized these methods to create image-reconstructed 3D models of primary PVS to study hemodynamic changes associated with stenosis and subsequent intervention in three patients. We demonstrated how PIV and CFD analyses, two common experimental modalities for flow analysis, can be instrumental for studying and quantifying biomechanical properties associated with congenital PVS. The velocity results from CFD analyses aligned well with the PIV results, demonstrating the potential of the simultaneous use of *in silico* and *in vitro* platforms for studying hemodynamic changes associated with cardiovascular interventions.

Furthermore, we demonstrated how CFD analysis using patient-specific *in vivo* parameters can be used to predict interventional outcomes through the WSS calculations. Recently published data indicated healthy levels of venous WSS approximated at 10 dyn/cm^2^, and neointimal hyperplasia levels are observed at 20–50 dyn/cm^2^ (12). In CFD studies here, 35 dyn/cm^2^ was chosen as an average upper limit of normal for venous WSS. Our CFD results demonstrated that TAWSS levels were above the upper limit of normal in several locations in the pre-catheterization models for all patients, predicting further stenosis progression. In the post-catheterization models, TAWSS levels decreased at the venoatrial junction; however, isolated areas of elevated TAWSS in the stent were still observed. Notably, our results suggested that TAWSS levels remain high at the bifurcation region, stented region, and high curvature areas for patients #1, #2, and #3, respectively. These results suggest that these patients may continue to have disease progression despite endovascular intervention, as frequently observed clinically. The outcomes from this study, therefore, highlight the need for improved intervention practices to reach desired WSS goals in patients with PVS. Although stent diameter is often limited by vascular compliance, other aspects of intervention may be altered by CFD and PIV prediction, including stent location and need for surgical intervention.

Future opportunities exist to further study altered hemodynamics and biomechanics of PVS using patient-specific modeling. 3D PIV methods could be employed to better recapitulate the hemodynamic behavior *in vitro* or *ex vivo*. Furthermore, as previously demonstrated in PVS, PAA, and tetralogy of Fallot with MAPCAs, 3D bioprinted models could be developed, using hydrogel-based biomaterials and a variety of cell types, and used to study cellular and genetic responses to venous stenosis (29, 41, 45–47). Using 3D bioprinted models, spatial transcriptomics and/or proteomic analyses could be highly informative tools to study genetic implications associated with the altered cellular microenvironment observed in primary PVS.

Several limitations and challenges exist in this study that could be addressed in future works. The resolution of CT images was determined to be adequate for creating 3D models; however, models with a high degree of stenosis, as seen in patient #2 with single-ventricle pathology, had very little contrast passthrough and a higher subjective degree of error when segmenting. Given the peristaltic flow profile of the pump used in the benchtop perfusion setup, the pediatric cardiac cycle velocity vs. time profile could not be simulated but was estimated using the determined average flow rate. PDMS has been shown to be an effective optically clear polymer to be used in PIV experiments; however, it is unable to precisely replicate the biomechanical properties of the congenital pulmonary veins. Moreover, the ability to resolve instantaneous WSS from 2D PIV is limited, and here, PIV results underpredict instantaneous and time-averaged WSS results obtained from CFD. Based on the CFD results, the velocity profile exhibits a steep slope, and the boundary layer thickness is only 0.2 mm. Although high-resolution PIV was employed to measure the flow, the vector spacing was approximately 0.12 mm, resulting in only two vectors within the boundary layer, which is insufficient to accurately capture the high-velocity gradient. Moreover, PIV uses a finite interrogation window, with an interrogation window of 64 pixels, ∼0.48 mm, used in these experiments, resulting in averaged values of vectors near the wall in the boundary layer. Consequently, the PIV technique cannot accurately determine the WSS values in the stenosis. Nonetheless, the inlet profile can be utilized to calibrate CFD simulations.

## 5 Conclusions

Congenital PVS is an ongoing clinical challenge for pediatric interventional cardiologists due to significant disease heterogeneity and progression despite intervention. 3D printing has been shown to be a useful technique for facing the challenge of modeling cardiovascular disease with considerable heterogeneity. In primary PVS, disturbed WSS is suggested to underlie disease pathogenesis through neointimal hyperplasia and venous fibrosis. Integrated CFD-PIV techniques continue to be an instrumental approach to model cardiovascular conditions with hemodynamic alterations. In this paper, using patient-specific 3D printed *in vitro* models we present how the CFD and PIV modalities can be used in parallel to simulate biomechanical changes in PVS. The obtained data can, in turn, be used to predict regions of disease progression, hence enabling more effective interventional procedures or therapies. Future studies are needed to further understand potential uses of patient-specific modeling using PIV, CFD, 3D bioprinting, and cellularization to improve outcomes for patients with PVS.

## Data Availability

The original contributions presented in the study are included in the paper/**Supplementary Material**, further inquiries can be directed to the corresponding authors.

## Supplementary material

The Supplementary Material for this paper can be found online at: https://www.frontiersin.org/articles/10.3389/fcvm.2024.1432784/full#supplementary-material.
