# Supplementary material for: Patient-specific 3D in vitro modeling and fluid dynamic analysis of primary pulmonary vein stenosis
Source: Front Cardiovasc Med. 2024 Jul 4;11:1432784. doi: 10.3389/fcvm.2024.1432784 (PMC11254695; doi:10.3389/fcvm.2024.1432784)
Supplement: Supplementary file 4 [file Datasheet1.docx]

# Supplemental Information

**Patient 1 - LLPV**

**
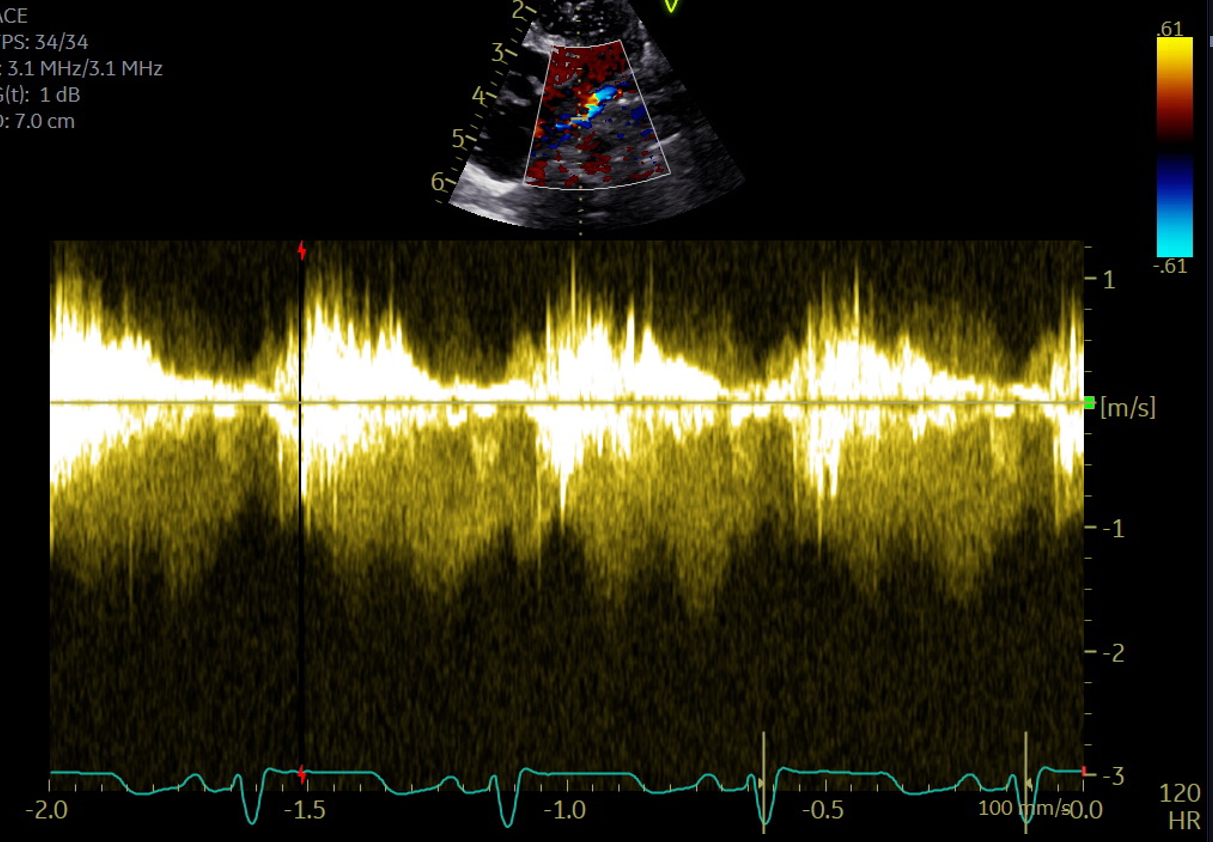

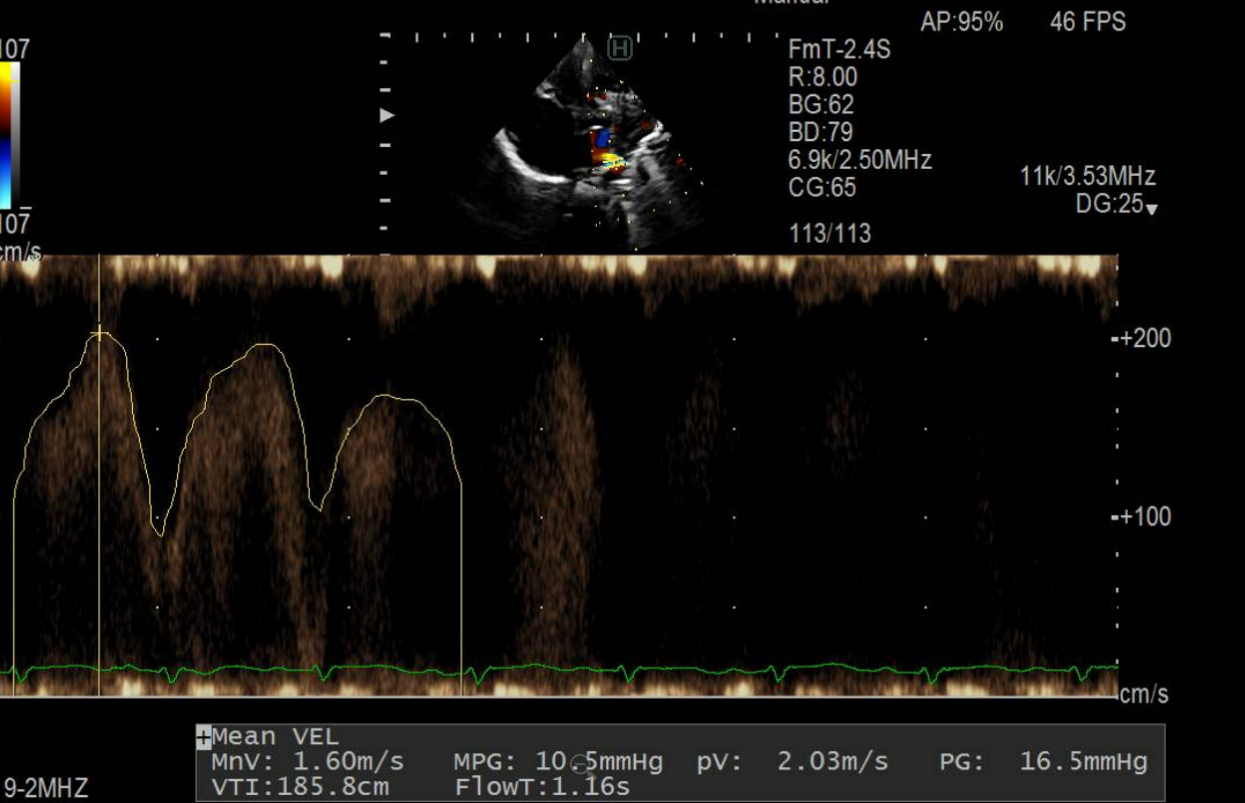
 Pre Cath Post Cath**

**Patient 2 - LLPV**

**Pre Cath Post Cath**
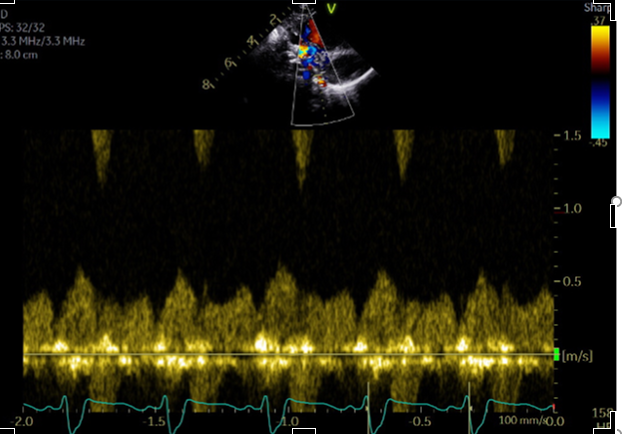

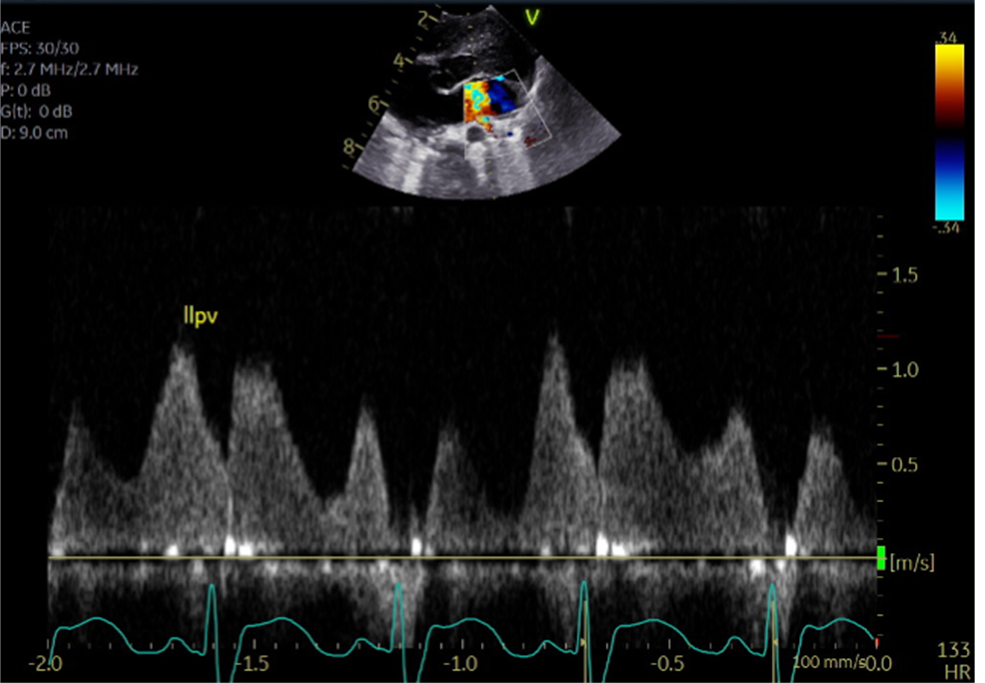


**Patient 3 - RUPV**

**Pre Cath Post Cath**


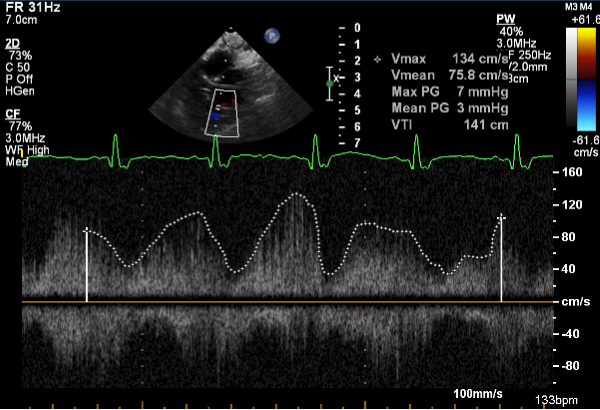

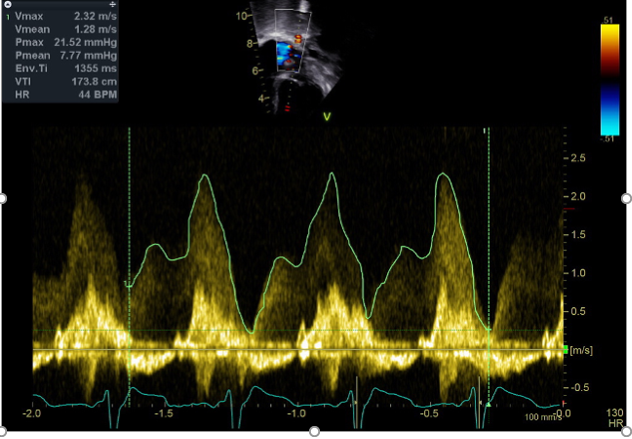


**SI Figure 1**: **Echocardiogram Data**: Pulse-doppler echocardiogram data for all patients pre- and post-catheterization.

**SI Table 1**: **List of pulmonary vein measurements**: Pulmonary vein measurements including determination of stenosis percentage in pre- and post-catheterization models.

| Patient # |  | Pre Cath | | | Post Cath |
| --- | --- | --- | --- | --- | --- |
|  | Pulmonary Vein | Stenosis Diameter (mm) | Stenosis Degree (%) | Distal Diameter (mm) | Stented Diameter (mm) |
| 1 | LUPV | 2.63 | 50 | 5.26 | 5.01 |
|  | RUPV | 3.12 | 41 | 5.29 | 5.88 |
|  | LLPV | 2.08 | 64 | 5.78 | 5.12 |
| 2 | LLPV | 0.40 | 83 | 2.35 | 3.72 |
| 3 | RUPV | 2.51 | 57 | 5.84 | 6.50 |

*LLPV: left lower pulmonary vein; RUPV: right upper pulmonary vein; LUPV: left upper pulmonary vein.*

**SI Video 1**: Patient 1 3D Contours of WSS During a Single Cardiac Cycle.

**SI Video 2**: Patient 2 3D Contours of WSS During a Single Cardiac Cycle.

**SI Video 3**: Patient 3 3D Contours of WSS During a Single Cardiac Cycle.
